# Specific Nucleotides in the Common Region of the Begomovirus Tomato Rugose Mosaic Virus (ToRMV) Are Responsible for the Negative Interference over Tomato Severe Rugose Virus (ToSRV) in Mixed Infection

**DOI:** 10.3390/v15102074

**Published:** 2023-10-11

**Authors:** Angélica M. Nogueira, Tarsiane M. C. Barbosa, Ayane F. F. Quadros, Anelise F. Orílio, Marcela C. J. Bigão, César A. D. Xavier, Camila G. Ferro, Francisco Murilo Zerbini

**Affiliations:** 1 Departamento de Fitopatologia, Universidade Federal de Viçosa, Viçosa 36570-900, MG, Brazil; angelica.nogueira@unesp.br (A.M.N.); tarsianemara@gmail.com (T.M.C.B.); ayane.quadros@ufv.br (A.F.F.Q.); anelise.orilio@ufv.br (A.F.O.); marcelacaarvalh@yahoo.com.br (M.C.J.B.); cdinizx@ncsu.edu (C.A.D.X.); cgfufv@hotmail.com (C.G.F.); 2 Instituto de Biotecnologia Aplicada à Agropecuária (BIOAGRO), Universidade Federal de Viçosa, Viçosa 36570-900, MG, Brazil; 3 Departamento de Proteção Vegetal, Faculdade de Ciências Agronômicas, Universidade Estadual Paulista (UNESP), Botucatu 18610-307, SP, Brazil; 4 Departamento de Entomologia e Acarologia, ESALQ, Universidade de São Paulo, Piracicaba 13418-900, SP, Brazil; 5 Department of Entomology and Plant Pathology, North Carolina State University, Raleigh, NC 27695, USA; 6 Departamento de Fitopatologia e Nematologia, ESALQ, Universidade de São Paulo, Piracicaba 13418-900, SP, Brazil

**Keywords:** geminiviruses, begomoviruses, infectivity, viral replication, *Rep* gene

## Abstract

Mixed infection between two or more begomoviruses is commonly found in tomato fields and can affect disease outcomes by increasing symptom severity and viral accumulation compared with single infection. Viruses that affect tomato include tomato severe rugose virus (ToSRV) and tomato rugose mosaic virus (ToRMV). Previous work showed that in mixed infection, ToRMV negatively affects the infectivity and accumulation of ToSRV. ToSRV and ToRMV share a high degree of sequence identity, including *cis*-elements in the common region (CR) and their specific recognition sites (iteron-related domain, IRD) within the *Rep* gene. Here, we investigated if divergent sites in the CR and IRD are involved in the interaction between these two begomoviruses. ToSRV clones were constructed containing the same nucleotides as ToRMV in the CR (ToSRV-A_(ToR:CR)_), IRD (ToSRV-A_(ToR:IRD)_) and in both regions (ToSRV-A_(ToR:CR+IRD)_). When plants were co-inoculated with ToRMV and ToSRV-A_(ToR:IRD)_, the infectivity and accumulation of ToSRV were negatively affected. In mixed inoculation of ToRMV with ToSRV-A_(ToR:CR)_, high infectivity of both viruses and high DNA accumulation of ToSRV-A_(ToR:CR)_ were observed. A decrease in viral accumulation was observed in plants inoculated with ToSRV-A_(ToR:CR+IRD)_. These results indicate that differences in the CR, but not the IRD, are responsible for the negative interference of ToRMV on ToSRV.

## 1. Introduction

The genus *Begomovirus* (family *Geminiviridae*) comprises plant viruses with a genome consisting of one (monopartite) or two (bipartite) single-stranded circular DNA molecules encapsidated separately by a single structural protein in twinned icosahedral particles [1]. In bipartite begomoviruses, the genomic components are referred to as DNA-A and DNA-B [2,3,4]. Genes in DNA-A encode proteins involved in replication, suppression of host defense responses and particle formation, while those in DNA-B encode proteins involved in viral movement throughout the plant and suppression of host defense responses [3,4]. Cognate DNA-A and DNA-B components share low nucleotide sequence identity, except for a region of approximately 200 nucleotides within the intergenic region, denominated as common region (CR), where identity is >85% [5].

Replication of begomoviruses occurs in the nuclei of infected cells by rolling circle and recombination-mediated mechanisms [6,7,8]. The virus-encoded Rep protein is essential for initiating replication [4,9]. Rep is a multifunctional protein with four highly conserved motifs in its N-terminal region [9,10]. Motif I (FLTY) is required for binding to dsDNA [11,12]; motif II (HLH) is involved in binding to metal ions; motif III contains the endonuclease activity responsible for cleavage at the origin of replication and binding at the resulting 3′-OH [10,13,14]; and motif IV (geminivirus Rep sequence, GRS), located between motifs II and III, is necessary for the initiation of rolling-circle replication and the establishment of viral infection [15].

To initiate replication, the Rep protein binds in a sequence-specific manner to the CR [9,16]. This region contains an origin of replication (ORI) structured in a conserved stem-loop, with the loop containing the invariant nonanucleotide 5′-TAATATT//AC-3′ [2] that is cleaved by Rep to generate the 3′-OH for the initiation of DNA synthesis [2,6,7]. The CR also contains iterated *cis*-elements (direct and inverted repeats) called iterons, which are presumably recognized by the iteron-related domain (IRD) of Rep to promote its binding to the CR [17,18]. In the original model, the IRD of begomovirus Rep proteins comprised six to eight amino acids and, together with motif I, formed the core of a hypothetical DNA-binding domain [12].

Generally, begomoviruses with identical iteron sequences encode Rep proteins with similar IRDs [12]. A correspondence between IRD and iteron sequences is found by the nucleotide–amino acid pairing that occurs between the iteron nucleotide N1 and the eighth amino acid of the IRD, N2 with the sixth amino acid and N3 with the first or third amino acids [12,19]. These specific amino acid residues within the IRD, which are important for iteron recognition, are named DNA binding specificity determinants (SPDs) [12,19]. Additional SPDs important for iteron binding have been identified in the vicinity of motif II of Rep [20], positioned at residues 10 and 12 ahead of the HuH core of the latter motif. Some SPDs may confer greater flexibility to Rep and allow the recognition of non-cognate, divergent iteron sequences [21].

Due to its site-specificity, the Rep protein efficiently recognizes cognate DNA-A and DNA-B components (which always have identical iterons). However, in the field, mixed infection between two or more begomoviruses often occurs, providing the opportunity to generate new viruses by the exchange of genomic components. The viability of these reassortants (or pseudorecombinants) will depend on the conservation of iteron sequences and of the amino acid sequence of the Rep protein within the IRD and the SPDs nearby to motif II, which control the affinity of the Rep by specific DNA sequences [12,19]. 

Tomato rugose mosaic virus (ToRMV) and tomato severe rugose virus (ToSRV) belong to a complex of bipartite begomoviruses that infect tomato in Brazil [22]. These viruses possess the same iteron core and identical SPDs, in addition to almost identical DNA-B sequences (98.2% identity). Silva et al. [23] demonstrated that ToRMV and ToSRV form viable pseudorecombinants in all possible combinations of single and mixed infections in tomato under experimental conditions. However, there was a preferential detection of ToRMV DNA-A and DNA-B in relation to ToSRV DNA-A and DNA-B, and the accumulation of ToSRV in mixed infection was reduced compared with that in single infection. Thus, not only is there no synergism between these two viruses, but ToRMV may negatively interfere with ToSRV accumulation.

Comparing the CR and Rep sequences of ToRMV and ToSRV, Silva et al. [23] found nine nucleotide differences in their CRs and different amino acids at positions 2 and 3 of the IRD (Thr/Pro and Arg/Lys for ToRMV/ToSRV, respectively). It is possible that one or more of these CR nucleotides and/or IRD amino acids play a role in the preferential replication of ToRMV in relation to ToSRV in a mixed infection.

Different mechanisms can favor the replication of one virus in relation to the other. The mechanisms responsible for the replication process of two or more begomoviruses present in the same cell are poorly understood. In this study, we evaluated the effect of nucleotide changes in the CR and in the region corresponding to the Rep IRD on ToSRV infectivity and accumulation. Our results indicate that the differences in the CR, but not in the IRD, are responsible for the negative interference of ToRMV on ToSRV.

## 2. Materials and Methods

### 2.1. Viral Isolates and Re-Sequencing of Isolates

Infectious clones corresponding to DNA-A and DNA-B of the viral isolates ToRMV-[BR:Ub1:96] [24] and ToSRV-[BR:PG1:Pep:03] [25] were used. These are the same clones used in the study of Silva et al. [23]. 

For confirmation of the sequences of the infectious clones, complete genome units were obtained by excision with BamHI (ToSRV DNA-A), EcoRI (ToRMV DNA-A) and KpnI (DNA-B from both viruses). After purification, these fragments were ligated into the pBLUESCRIPT-KS+ vector (Agilent, Santa Clara, CA, USA), previously linearized with the same enzymes. Recombinant plasmids were used for transformation into *Escherichia coli* by electroporation, and three clones of each genomic component were sent for commercial sequencing by primer walking (Macrogen Inc., Seoul, Republic of Korea). Sequences were assembled using DNA Baser v. 3.5 (Heracle Biosoft, Mioveni, Romania) and organized to begin at the nicking site in the invariant nonanucleotide at the origin of replication (5′-TAATATT//AC-3′). Pairwise sequence comparisons of DNA-A and DNA-B, CRs and ORFs between ToRMV and ToSRV were performed using Sequence Demarcation Tool (SDT) v.1.2 [26] with the MUSCLE alignment option [27]. 

### 2.2. Construction of Infectious Clones

**Figure 1:**
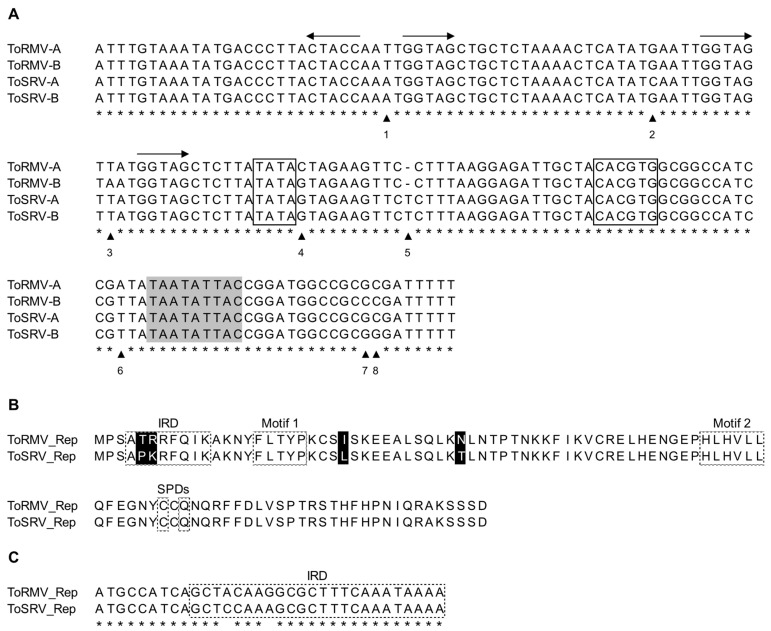
(**A**) Alignment of the nucleotide sequences of the common region (CR) of the DNA-A and DNA-B of tomato rugose mosaic virus (ToRMV) and tomato severe rugose virus (ToSRV). The arrowheads and numbers indicate the eight divergent positions among the CRs of the four components. Asterisks represent conserved nucleotides among the aligned sequences. The horizontal arrows indicate the direction of the iterons. The TATA box and the G-box are boxed. The conserved nonanucleotide at the origin of replication is indicated in light gray. (**B**) Alignment of the amino acid sequences of the N-terminal portion of the Rep proteins of ToRMV and ToSRV. Divergent amino acids are highlighted in black. The iteron-related domain (IRD), specificity determinants (SPDs) and protein motifs are boxed with dashed lines. (**C**) Alignment of the nucleotide sequences of the region corresponding to the IRDs of ToRMV and ToSRV. Identical nucleotides are indicated by an asterisk.

After re-sequencing of the ToRMV and ToSRV infectious clones, nucleotide differences were observed at eight positions in the CR and amino acid differences were observed in two positions within the IRD of the Rep protein (Figure 1). 

**Figure 2:**
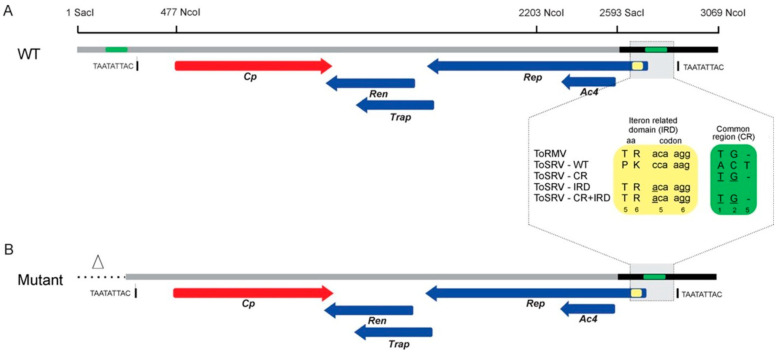
Schematics of the construction of the ToSRV clones containing the ToRMV nucleotides at the divergent positions in the CR and the *Rep* gene IRD. Altered nucleotides in the CR and IRD are indicated by the green and yellow boxes, respectively. In the CR (green box), the numbers 1, 2 and 5 represent divergent positions, and underlined bases indicate the changes. In the IRD (yellow box), the numbers 5 and 6 represent the amino acids encoded by the fifth and sixth codons of the *Rep* gene, and underlined bases indicate the changes. (**A**) Representation of the ToSRV DNA-A infectious clone containing a full-length copy of the DNA-A (2593 nt, gray line) plus a 476 bp tandem fragment (black line) containing the origin of replication. (**B**) Representation of the ToSRV DNA-A infectious clone after the deletion of the repeated region (triangle and dotted line). The nonanucleotide at the origin of replication (TAATATTAC) is indicated. The red and blue arrows represent the viral genes and the direction in which they are transcribed (viral and complementary sense, respectively).

To verify if these differences are responsible for the reduced accumulation of ToSRV and the predominance of ToRMV over ToSRV in a mixed infection, a full-length clone of the ToSRV DNA-A exchanging the nucleotides at the divergent positions in the CR and the IRD by those of ToRMV DNA-A (named ToSRV-A_(ToR:CR+IRD)_) was synthesized using the GenPlus service from the company GenScript (Piscataway, NJ, USA) (Figure 2). In the ToSRV DNA-A CR, the adenine at position 1 (Figure 1) was replaced by thymine, the cytosine at position 2 by a guanine and the thymine at position 5 was deleted. In the *Rep* gene IRD, the first base of the fifth codon (cytosine) was replaced by adenine and the second base of the sixth codon (adenine) was replaced by guanine. These two substitutions altered the amino acid sequence of the Rep protein (proline to threonine and lysine to arginine, respectively) (Figure 2). As the Rep protein is expressed from the virion complementary strand (synthesized during viral DNA replication), the bases replaced on the virion strand of the infectious clone sequence are complementary bases; thus, in the region corresponding to the fifth codon of *Rep* in the virion strand, a guanine was replaced by thymine, and in the sixth, a thymine by a cytosine. The synthesized DNA-A was cloned on the pUC57 vector, and Sanger sequencing was performed to confirm that only the nucleotides of interest were inserted. 

To construct the infectious clone, the complete sequence of ToSRV-A_(ToR:CR+IRD)_ was subjected to in silico restriction analysis using the program ApE (A plasmid Editor) v. 2.0.53 [28]. The restriction enzyme SacI, which cleaves at only one point in the genome and is located closer to the origin of replication, was chosen to simulate the site that would be used for cloning the full-length ToSRV-A_(ToR:CR+IRD)_ (2593 bp). To generate the partially redundant portion of the ToSRV clone that must contain the origin of replication, we used SacI and NcoI to generate a 476 bp fragment (SacI2593 to NcoI3069). This fragment also contains the region where the ToRMV nucleotides were inserted (represented as green rectangles in Figure 2A). The presence of the same region where the ToRMV nucleotides were inserted in both the 2593 bp and 476 bp fragments would hinder the site-specific mutagenesis process to be performed subsequently, because the primer containing the ToRMV nucleotides could anneal in only one of two regions during PCR. Considering that, for a clone to be infectious, it needs to have no more than two origins of replication, the repeated region where the ToRMV nucleotides were inserted and the one immediately upstream of it were excluded from the fragment corresponding to one copy of the viral genome, generating an infectious clone with 2829 bp instead of 3069 (Figure 2B). After synthesis, the fragment was cloned into the pUC57 vector using the blunt end enzyme EcoRV. 

Additional clones containing only the CR (ToSRV-A_(ToR:CR)_) or only the IRD ToRMV nucleotides (ToSRV-A_(ToR:IRD)_) were constructed by site-directed mutagenesis (also by GenScript), using ToSRV-A_(ToR:CR+IRD)_ as a template. To construct ToSRV-A_(ToR:CR)_, the two ToRMV nucleotides in the *Rep* gene were reversed to the original sequence. To construct ToSRV-A_(ToR:IRD)_, the three ToRMV nucleotides in the CR were reversed to the original sequence (Figure 2). Confirmation of all clones was performed by Sanger sequencing. 

### 2.3. Inoculation of Plants

Tomato (*Solanum lycopersicum* cv. Santa Clara) seedlings were inoculated by biolistics [29] using 10 mg of each genomic component (DNA-A and DNA-B) of the wild-type (wt) ToRMV and ToSRV infectious clones, and the clones ToSRV-A_(ToR:CR)_, ToSRV-A_(ToR:IRD)_ and ToSRV-A_(ToR:CR+IRD)_ in single (ToRMV-A+B; ToSRV-A_(wt)_+B; ToSRV-A_(ToR:CR)_+B; ToSRV-A_(ToR:IRD)_+B; and ToSRV-A_(ToR:CR+IRD)_+B) and mixed inoculations (ToSRV-A_(wt)_+B + ToRMV-A+B; ToSRV-A_(ToR:CR)_+B + ToRMV-A+B; ToSRV-A_(ToR:IRD)_+B + ToRMV-A+B; and ToSRV-A_(ToR:CR+IRD)_+B + ToRMV-A+B). The ToSRV wt DNA-B was always inoculated with the ToSRV clones in single and mixed inoculations. Tomato plants were also bombarded with DNA-free gold particles as a negative control. Ten plants were inoculated per treatment and four independent experiments were performed, with a total of 38–40 inoculated plants per treatment, as some plants were lost in the third and fourth experiments. Plants were kept in a greenhouse and expression of symptoms was evaluated for up to 35 days after inoculation (dai).

### 2.4. Detection and Quantification of Genomic Components

Total DNA from all plants was extracted from the second youngest leaf at 28 dai as described by Doyle and Doyle [30]. For confirmation of single infection, the DNA-A of ToRMV and ToSRV was detected by PCR using primers ToRMV-A_(For)_ (5′-CAG TAG TTG CCT TCG AAT TGA AG-3′), ToRMV-A_(Rev)_ (5′-CAC GTG TAG CAA TCT CCT TAA AGG-3′), ToSRV-A_(For)_ (5′-CAG TAG TTG CCC TCA AAT TGA AG-3′) and ToSRV-A_(Rev)_ (5′-CAC GTG TAG CAA TCT CCT TAA AGA G-3′). Reactions contained 1 μL of total DNA, 0.4 μM of each primer, 1x Reaction Buffer Complete (Celco), 0.2 mM of each dNTP and 1.25 U of Taq DNA Polymerase (5 U/μL) (Celco) in a final volume of 25 μL. PCR parameters consisted of an initial denaturation step at 95 °C for 2 min; followed by 38 cycles at 95 °C for 1 min, 66 °C for 1 min and 72 °C for 1 min; followed by a final extension at 72 °C for 10 min. PCR products were separated by 1% agarose gel electrophoresis and stained with ethidium bromide. Confirmation of mixed infection was performed by amplification of the complete viral genome by rolling circle amplification (RCA) [31] followed by cleavage with specific restriction enzymes for the detection of each component (RCA-RFLP), as described by Silva et al. [23]. 

After confirmation of single or mixed infections in the plants, the positive plants were selected for analysis of ToSRV and ToRMV DNA-A accumulation. In mixed infection, only plants in which ToSRV DNA-A was detected simultaneously with ToRMV DNA-A were selected. Accumulation was assessed by absolute quantification using quantitative real-time PCR (qPCR), performed in a StepOnePlus system (Applied Biosystems) using 100 ng of total DNA and primers qToSRV-A_(For)_ (5′-AAA GTA AAG TGA TTG TCT GTG G-3′), qToSRV-A_(Rev)_ (5′-GCC GTT CAA CAA ATT GGG-3′), qToRMV-A_(For)_ (5′-CTT AGA TGT ACT AGC CAT GTG G-3′) and qToRMV-A_(Rev)_ (5′-CCT TTA ATT TTA TTG AAA ATA ATT TTG GC-3′). All reactions were performed in technical triplicates for each biological replication. Thermocycling conditions consisted of an initial denaturation step of 95 °C for 3 min followed by 40 cycles of 95 °C for 10 s and 66 °C for 30 s, with a final dissociation step. Viral accumulation was determined by interpolation of the Ct values of each tested sample within the standard curves for ToRMV and ToSRV. To construct the standard curves, serial dilutions (5 × 10^4^ to 5 × 10^9^ copies) were prepared from known quantities of plasmids containing a single copy of the genomic component of each virus. Quantifications of the plasmid DNA used to construct the standard curves and the total DNA samples were performed using a Nanodrop 2000c (ThermoFisher Scientific, Waltham, MA, USA).

### 2.5. Sequence Comparisons

Multiple sequence alignments were prepared for the CRs and the Rep of ToSRV isolates using the MUSCLE alignment option in MEGA11 [32]. 

### 2.6. Analysis of Structural Properties

The AlphaFold2 program [33] was used to predict the tertiary structure of the Rep of ToRMV, ToSRV and ToSRV_(ToR:IRD)_. After 3D structure prediction, the RMSD and TM-score were calculated using the mTM-align tool [34]. Structures were visualized using RCSB PDB Mol* Viewer v2.6.0.

### 2.7. Statistical Analysis

Data were analyzed by ANOVA with a significance level of *p* ≤ 0.05 followed by Fisher’s least significant difference (LSD) test to separate means into homogeneous groups. The analyses were performed using R version 4.2.2 [35].

## 3. Results

### 3.1. Effects of Nucleotide Changes on ToSRV and ToRMV Infectivity

To verify whether the nucleotide differences observed in the CR and IRD of the Rep protein are responsible for the reduction of ToSRV accumulation and for the preferential detection of ToRMV in mixed infection with ToSRV, infectious clones of ToSRV DNA-A containing the same sequence of the ToRMV DNA-A in the CR (ToSRV-A_(ToR:CR)_), the IRD (ToSRV-A_(ToR:IRD)_) and in both regions (ToSRV-A_(ToR:CR+IRD)_) were inoculated in tomato plants. Treatments consisted of single infection, where each clone was inoculated along with wt ToSRV DNA-B, and mixed infection, where the clones were inoculated along with wt ToSRV DNA-B plus wt ToRMV DNA-A and DNA-B. Symptoms started to appear at 10–14 dpi and consisted of yellow mosaic and leaf distortion in all treatments, with no differences among them (Supplementary Figure S1). Thus, based on symptoms, no synergistic interaction was observed between the two viruses. All subsequent analyses were performed at 28 dai since there were no differences in symptoms between 28 and 35 dai.

The results of the infectivity test are presented in Table 1 and Supplementary Table S2. The overall infectivity rate of single infection was statistically similar for all treatments, ranging from 35% to 57.5%. These results indicate that wt and mutant ToSRV and wt ToRMV infect tomatoes in single infection with equivalent efficiency.

Mixed infection was screened by RCA-RFLP to verify the presence of each inoculated DNA component (one representative of each DNA-A and DNA-B combination of viruses detected in each treatment is shown in Supplementary Figure S2). The results for each treatment were consistent in all experiments, with only a few discrepancies observed for detection of wt DNA-B components in the ToSRV-A_(ToR:CR)_ treatment (Table 1). It is also noteworthy that the wt ToSRV DNA-B component was detected at a much lower percentage than the wt ToRMV DNA-B component in all treatments (Table 1).

Considering only the DNA-A detection, when wt ToRMV was inoculated with wt ToSRV, the ToRMV DNA-A (henceforth referred to as ToR-A) was detected at a higher rate compared to the ToSRV DNA-A (ToS-A) (37.5% and 27.5% for ToR-A and ToS-A, respectively). However, in most plants, the DNA-A component of each virus was detected alone, with only a small number of plants infected by the two components (four plants with ToS-A + ToR-A vs. seven plants with ToS-A alone and eleven with ToR-A alone). Equivalent results were obtained for the ToSRV-A_(ToR:IRD)_ clone, with 27.5% and 17.5% of the plants infected with ToR-A and ToS-A_(ToR:IRD)_, respectively, but with both components being detected in only one plant vs. six plants with ToS-A_(ToR:IRD)_ alone and ten plants with ToR-A alone. Strikingly, for the ToSRV_(ToR:CR)_ clone, the ToR-A and ToS-A_(ToR:CR)_ components were detected in a similar number of plants (45% and 50%, respectively) and most plants were infected by the two components (fifteen plants with both components vs. five with ToS-A_(CR)_ alone and three with ToR-A alone). With the ToSRV_(ToR:CR+IRD)_ clone, both components were also detected at similar rates (28.9% and 26.3% for ToR-A and ToS-A_(ToR:CR+IRD)_, respectively), with six plants infected by the two components, four with ToS-A_(ToR:CR+IRD)_ alone and five with ToR-A alone (Table 1). Statistical analysis was performed to compare the infectivity of ToSRV-A_(wt)_ in single infection with the same component (or its mutants) in mixed infection with ToRMV (Supplementary Table S2). The results confirmed the negative effect of ToRMV on ToSRV, with a statistically significant decrease in the detection of ToS-A_(wt)_, ToS-A_(ToR:IRD)_ and ToS-A_(ToR:CR+IRD)_ in the presence of ToRMV. On the other hand, there was no statistically significant difference in the detection of the ToS-A_(ToR:CR)_ mutant in the presence of ToRMV, compared to single infection by ToSRV. Together, these results indicate that the nucleotide differences in the CR, but not the amino acid differences in the IRD, are responsible for the prevalence of ToRMV components over ToSRV components. 

### 3.2. Effects of Nucleotide Changes on ToSRV and ToRMV Accumulation

To verify the accumulation of ToRMV and ToSRV DNA-A in single and mixed infections, quantitative real-time PCR (qPCR) was performed using virus-specific DNA-A primers. For the analysis of plants with mixed infection, quantification of viral accumulation was performed only for plants in which DNA-A components of both viruses were confirmed to be present. Since this was the case for only one plant in the ToSRV-A_(ToR:IRD)_ + ToRMV treatment (Table 1), this treatment was not included in the analysis. 

**Figure 3:**
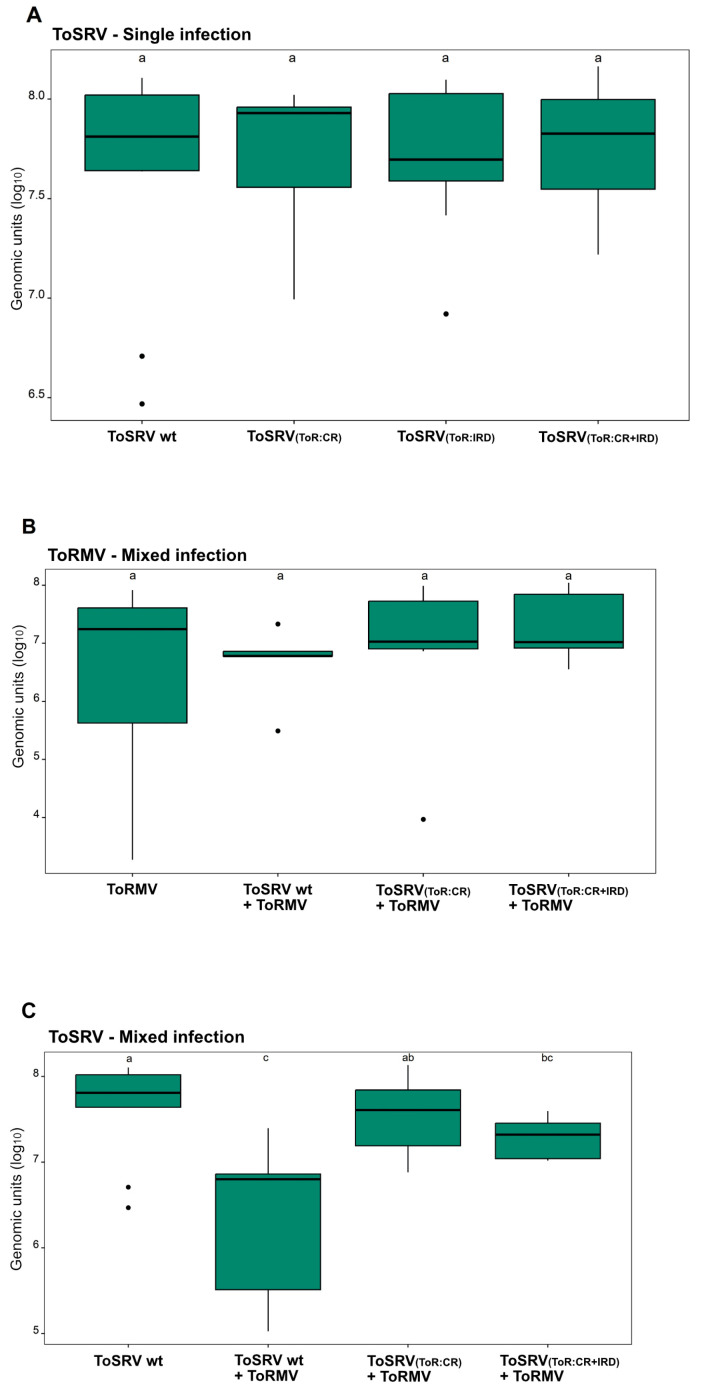
Accumulation of tomato rugose mosaic virus (ToRMV) and tomato severe rugose virus (ToSRV_(wt)_, ToSRV-A_(ToR:CR)_, ToSRV-A_(ToR:IRD)_ and ToSRV-A_(ToR:CR+IRD)_) DNA-A components in (**A**) single and (**B**,**C**) mixed infections in tomato plants. Absolute quantification of viral DNA was performed at 28 days post-inoculation. Boxplots correspond to viral accumulation, presented as the logarithm of the number of molecules. Dots indicate outliers. Means were compared using Fisher’s least significant difference (LSD) test. Letters represent statistically significant differences (*p* < 0.05), and error bars indicate the standard deviation.

In single infection, no differences in ToS-A accumulation were observed for the wt ToSRV, ToSRV-A_(ToR:CR)_, ToSRV-A_(ToR:IRD)_ and ToSRV-A_(ToR:CR+IRD)_ treatments (Figure 3A), confirming that all three mutant ToSRV DNA-A clones are capable of replicating at equivalent wt levels. ToRMV was also quantified in single infection and in mixed infection with ToSRV, with no differences in its accumulation among treatments (Figure 3B). When wt ToSRV was quantified in mixed infection with ToRMV, the accumulation of ToSRV was significantly reduced (Figure 3C), confirming the previous report by Silva et al. [23]. Strikingly, in the plants inoculated with ToSRV-A_(ToR:CR)_ and ToRMV, the accumulation of ToS-A_(ToR:CR)_ was statistically equivalent to that of wt ToSRV in single infection and corresponded to an 80% increase in relation to wt ToSRV in mixed infection with ToRMV (Figure 3C). The accumulation of ToSRV-A_(ToR:CR+IRD)_ in mixed infected plants corresponded to 50% of that of wt ToSRV in single infection but, interestingly, was statistically equivalent to that of ToSRV_(ToR:CR)_ (Figure 3C). The results of viral DNA accumulation are consistent with the infectivity assay (Table 1), indicating that the nucleotide differences in the CR are responsible for the negative interference of ToRMV on ToSRV.

### 3.3. Nucleotide and Amino Acid Differences among ToSRV Isolates

We compared ToSRV-[BR:PG1:Pep:03] to a total of 144 ToSRV isolates from different hosts to see how common the observed variations in the CR and IRD are among isolates of the virus. Analyzing the IRD nucleotide sequence, we found that only five of these isolates contained a single variable nucleotide and that only two out of these five isolates lack cytosine as the first base of the fifth Rep codon (like ToSRV-[BR:PG1:Pep:03] does) (Supplementary Figure S3). For the CR region, it was observed that the adenine found in position 1 of ToSRV-[BR:PG1:Pep:03] is also present in six other isolates obtained from beans, soybeans and tomatoes (BR:ITA1274:14, BR:SOITA1014:14, BR:DF607, BR:Pip1792:03, BR:G2 and BR:G3). All other isolates have a thymine at this position, which is the same as what is found in ToRMV. At position 2, the ToSRV-[BR:PG1:Pep:03] isolate has a cytosine but every other isolate, including the ToRMV isolate, has a guanine. At position 5, 3.5% of the isolates contain cytosine, 45.1% of the isolates resemble ToRMV without a nucleotide in this region and 51.4% of the isolates resemble ToSRV-[BR:PG1:Pep:03] (Supplementary Figure S3).

### 3.4. Analysis of Structural Properties

**Figure 4:**
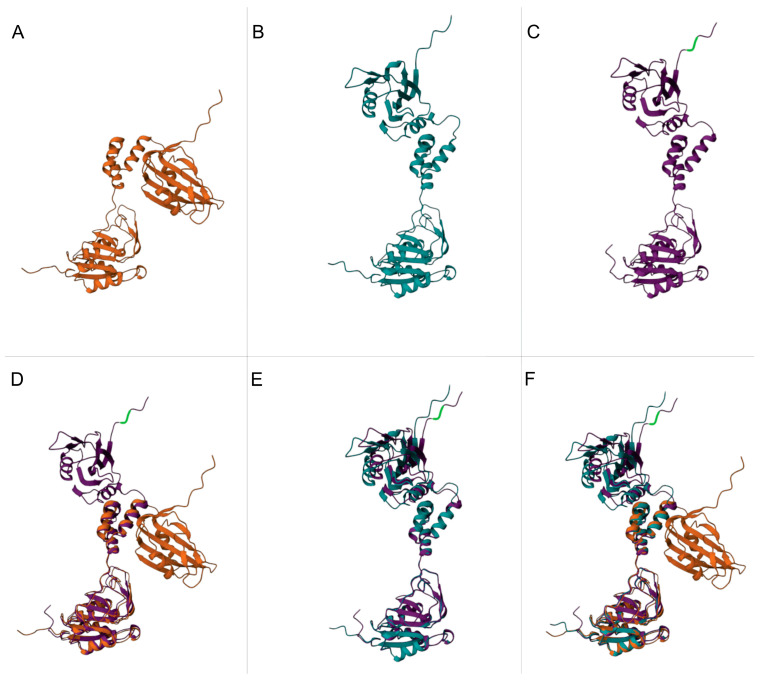
Rep protein tertiary structure prediction by Alphafold. (**A**) Tomato rugose mosaic virus (ToRMV) Rep protein (orange). (**B**) Tomato severe rugose virus (ToSRV) wild type (wt) Rep protein (blue). (**C**) ToSRV_(ToR:IRD)_ Rep protein (purple). (**D**) Overlay of ToRMV and ToSRV_(ToR:IRD)_ Rep proteins. (**E**) Overlay of wt ToSRV and ToSRV_(ToR:IRD)_ Rep proteins. (**F**) Overlay of ToRMV, wt ToSRV and ToSRV_(ToR:IRD)_ Rep proteins. The sites where nucleotides were changed in ToSRV_(ToR:IRD)_ are highlighted in green.

To investigate possible conformational differences in the structure of the Rep protein of ToRMV, ToSRV wt and ToSRV_(ToR:IRD)_ that could be related to the virus-specific recognition of the iterons, an in silico prediction was performed using the AlphaFold2 program. As mutations in the CR would not affect the structure of any virus-encoded protein, ToSRV_(ToR:CR)_ and ToSRV_(ToR:CR+IRD)_ were not considered in the modeling. All models generated by the program had high confidence metrics (Figure 4). The similarity measure provided by the TM-score value was 0.62 between wt ToRMV and ToSRV, and the same value was observed for the comparison between ToRMV and ToSRV_(ToR:IRD)_. The TM-score value for ToSRV_(ToR:IRD)_ in relation to wt ToSRV was 0.96. 

The ToSRV_(ToR:IRD)_ clone was evaluated with single changes: ToSRV_(Pro>Thr)_ and ToSRV_(Lys>Arg)_. ToSRV_(Pro>Thr)_ had a TM-score of 0.62, 0.94 and 0.98 in relation to ToRMV, wt ToSRV and ToSRV_(ToR:IRD)_, respectively. ToSRV_(Lys>Arg)_ yielded TM-scores of 0.62, 0.97 and 0.97 in relation to ToRMV, wt ToSRV and ToSRV_(ToR:IRD)_, respectively.

## 4. Discussion

The host range of viruses classified in the same family often overlap considerably. Moreover, viruses classified in the same genus usually share the same type of vector (such as whiteflies for begomoviruses). Thus, mixed infection by begomoviruses (genus *Begomovirus*) in the same plant are very common in nature [36,37,38]. In this co-infection process, viral fitness can be altered due to virus–virus or virus–plant interactions that may occur and influence viral replication and accumulation [39,40]. Nevertheless, the current understanding of the dynamics of mixed infection is limited, since the mechanisms that control the differences in viral accumulation and, consequently, the possible predominance of one virus over the other are unknown. In this study, we analyzed the interaction between ToSRV and ToRMV to better understand co-infection aspects. Our results indicate that the apparently minor differences in the common region (CR) sequence of these two begomoviruses can strongly influence their interaction.

Ribeiro et al. [41] and Silva et al. [23] suggested that ToRMV is a recombinant whose parental viruses are ToSRV and tomato chlorotic mottle virus (ToCMoV). Recombination analysis revealed that the ToRMV DNA-A contains a portion of the CR, including the iterons and nearly all of the *Rep* gene derived from ToSRV, and that ToRMV captured ToSRV DNA-B. The DNA-B components of ToRMV and ToSRV have 98.2% nucleotide sequence identity. 

Recombination and pseudorecombination are frequent and relevant mechanisms for the generation of genetic variability in begomoviruses [3,21,42,43]. Recombination sites are not randomly distributed in the genome: the origin of replication and the 5′-terminal portion of the Rep gene are recombination hot spots, frequently exchanged during replication [2,44]. The formation of viable pseudorecombinants is common among isolates of the same virus [45,46,47,48] and can also occur between distinct viruses that exhibit high identity in the CR sequences of the heterologous components [19,21,49,50,51,52]. Thus, the viral factors involved in replication can be transferred (by recombination) or shared (by pseudorecombination) between different viruses.

ToSRV clones containing the same nucleotides as ToRMV at divergent positions in the CR and IRD were constructed to determine whether these regions could improve ToSRV infectivity and replication efficiency when in mixed infection with ToRMV. Indeed, both parameters were improved in the case of ToSRV-A_(ToR:CR)_ but not ToSRV_(ToR:IRD)_. ToSRV-A_(ToR:CR+IRD)_ also had improved infectivity and accumulation, although not as significantly as ToSRV_(ToR:CR)_. Thus, the presence of T and G at CR positions 1 and 2 and a deletion at position 5 (numbered according to the alignment of Figure 1A) seems to confer an adaptive advantage to ToSRV isolates in tomato plants when in mixed infection with ToRMV. 

Recognition of the iterons by the Rep protein is a key event for binding of the Rep/REn complex to the origin of replication and, thus, for the initiation of viral replication [16,17,18]. According to the proposed model of recognition between Rep and the iterons (Arguello-Astorga and Ruiz-Medrano [12]), the sixth and eighth amino acids of the IRD (which are the same for ToRMV and ToSRV) control the binding efficiency of Rep to the viral genome, and the second and third amino acids (which diverge between ToRMV and ToSRV) are not supposed to dictate preference for specific iteron sequences. However, this model was based entirely on in silico analyses and functional analyses were not performed.

The amino acids that determine the high binding affinity of Rep to iterons have been little explored so far [53]. The exchange of only one amino acid residue at position 10 between Rep proteins from two tomato leaf curl New Delhi virus (ToLCNDV) isolates (equivalent to the amino acid residue at position 11 in Rep proteins of ToRMV and ToSRV) resulted in a shift in replication specificity and an increase in viral DNA accumulation [54]. The IRD domain contains a small β1 strand, which is an extension of the larger β2-strand including motif I; the β1-strand interacts with the small β5-strand that comprises the putative SPDs close to motif II. A mini β-sheet is formed by the interaction of β1- and β5-strands, and this structure is composed of a cluster of amino acid residues that recognizes specific dsDNA sequences (i.e., the iterons) in the plus-strand replication origin of geminivirus, as proposed by Campos-Olivas et al. [55] when analyzing the Rep of tomato yellow leaf curl virus (TYLCV). In β-sheet structures, hydrophobic amino acids are recurrent, whereas proline and charged amino acids (Arg, Lys, Glu and Asp) are underrepresented [56]. In ToRMV, the IRD has Thr and Arg amino acids at positions 2 and 3 of the domain, while ToSRV has Pro and Lys. Thus, it is not unreasonable to assume that the replacement of Pro by Thr in ToSRV_(ToR:IRD)_ would improve the recognition of the iterons, while the replacement of Lys by Arg would have a neutral effect. The fact that an improvement was not observed is puzzling. On the contrary, the phenotype of ToSRV_(ToR:CR+IRD)_ suggests that the IRD mutations had an adverse effect in the IRD in terms of iteron recognition. In addition, we observed that the mutations did not lead to conformational changes in the Rep protein. Thus, the mutations likely influence recognition affinity; although, how this would take place is currently unknown.

One hypothesis to explain these observations is that the ToSRV Rep could be more flexible in its ability to recognize iterons, whereas the ToRMV Rep would be more stringent. This hypothesis arises from analyses of the N-terminal region of the Rep of other begomoviruses that infect tomato, such as ToCMoV, tomato yellow spot virus (ToYSV) and tomato golden mosaic virus (TGMV). Despite having distinct IRD sequences, these viruses have the same amino acids as ToSRV at these divergent positions in the IRD [21]. Another possibility is that the ToRMV-derived CR sequence may favor replication, while the ToRMV-derived IRD sequence provides a disadvantage. To verify whether the ToRMV Rep IRD amino acids confer greater binding specificity, infectivity assays with ToRMV clones in which amino acids in the divergent IRD sites were replaced with the ToSRV-encoded amino acids should be conducted. It will also be interesting to determine if mixed infection with ToYSV or ToCMoV has the same effect negative on ToSRV and whether the adaptive changes observed in the ToSRV_(ToR:CR)_ mutant also occur in mixed infection with these viruses.

Complex interactions are observed in the coexistence of two or more viruses and can lead to changes in a number of characteristics, including the transmission efficiency [57], severity of symptoms [37], breakdown of resistance in cultivated hosts [58,59], cell tropism [60,61] and viral DNA titer [36,62]. Our results of viral load quantification indicated that ToSRV accumulation is drastically reduced in mixed infection with ToRMV. Interestingly, ToRMV also negatively interferes with the accumulation of ToYSV during the initial stages of infection in tomato [61]. A reduced rate of viral accumulation may be due to competition for host resources in mixed infection, resulting in less accumulation of one or both viruses compared to single infection [63]. On the other hand, beneficial effects of mixed infection involving begomoviruses have been reported for pepper plants infected with pepper huasteco yellow vein virus (PHYVV) and pepper golden mosaic virus (PepGMV) [37], and in cassava infected with African cassava mosaic virus (ACMV) and East African cassava mosaic virus (EACMV) [64]. In both cases, mixed infection resulted in higher viral concentrations of both viruses and more severe symptoms compared with single infection. Despite differences in viral accumulation, synergism was not identified in our investigation, with similar symptoms observed in single and mixed infections.

According to Martin and Elena [36], different individuals in a population might be viewed as unique participants in a biological game. The winner will be the one with the best strategy (highest fitness) and, as a result, will have the highest frequency in the population. Based on the infectivity of the genomic components and the accumulation of viral DNA, it is tempting to propose that the divergent nucleotides in the ToRMV CR make this virus a better competitor in the mixed infection with ToSRV due to more efficient binding of Rep to the viral DNA. However, it is not self-evident that an increase in the affinity of Rep for its binding sites in the CR would results in higher viral replication, because Rep represses its own expression by binding to the iterons associated with the TATA box. Consequently, a higher affinity for its cognate iterons could also have a negative effect on gene expression and protein synthesis. This possible trade-off between replication and gene repression has not been systematically studied.

In this context, an alternative explanation for the increase in ToSRV_(ToR-CR)_ replication could be sought in the multiple roles associated with the begomovirus CR. Besides the plus-strand replication origin, the CR also contains the minus-strand replication origin, which is involved in the generation of the complementary strand from the ssDNA by the DNA primase of the host. This (−)ori generates the dsDNA intermediate that transcribes the viral genes. The precise (−)ori nucleotide sequence has not been reported for any begomovirus to date, but it has been mapped very close to the 5′ end of the stem-loop element of the (+)ori [65]. Thus, it seems reasonable to assume that if the (−)ori of ToRMV and ToSRV function with different efficiencies because a mutation in a critical nucleotide occurred, the overall virus replication rate and the level of DNA accumulation could be altered in a mixed infection. Therefore, it could be hypothesized that differences at position 5 (and also at position 6, which was not changed in our study) between the CRs of ToRMV and ToSRV could potentially be responsible for the lower DNA accumulation of ToSRV in mixed infection with ToRMV.

Given the prevalence of ToSRV over ToRMV in the field, the disadvantage presented by ToSRV in comparison with ToRMV in mixed infection is somewhat contradictory [22,66,67]. Interestingly, most ToSRV field isolates share the same nucleotides as ToRMV at the three CR positions examined in our study. Therefore, it is possible that the isolate utilized in the construction of the infectious clone (BR:PG1:Pep:03) is an atypical ToSRV isolate. To determine whether the negative effect exerted by ToRMV prevails, interaction studies between ToRMV and other ToSRV isolates that do not contain nucleotide differences at these positions would be helpful.

As a final observation, the isolate ToSRV-[BR:PG1:Pep:03] was obtained from pepper rather than tomato. The differences observed in the CR sequence of ToSRV-[BR:PG1:Pep:03] may be the result of viral adaptation to a new host, and this adaptation may have incurred a fitness cost. Although ToSRV naturally infects tomato and pepper, infection in tomato is much more prevalent in the field. New studies comparing ToSRV isolates from various hosts may provide additional insights into viral evolution in terms of adaptation to new hosts and potential fitness effects.

## Figures and Tables

**Table 1:** Infectivity of tomato severe rugose virus (ToSRV) in single infection and in mixed infection with tomato rugose mosaic virus (ToRMV).

Treatment *	Genomic Component	Infected Plants ^$^		
ToSRV_(wt)_	ToS-A_(wt)_ ^#^	16/40 (40)		
ToRMV	ToR-A ^#^	14/40 (35) ^ns^		
ToSRV-A_(ToR:CR)_	ToS-A_(ToR:CR)_ ^#^	19/39 (48.7) ^ns^		
ToSRV-A_(ToR:IRD)_	ToS-A_(ToR:IRD)_ ^#^	23/40 (57.5) ^ns^		
ToSRV-A_(ToR:CR+IRD)_	ToS-A_(ToR:CR+IRD)_ ^#^	20/40 (50) ^ns^		
ToSRV-A_(ToR:CR)_ + ToRMV	ToS-A_(ToR:CR)_ + ToR-A ^&^	15/40 (37.5)	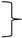	^ns^
	ToS-A_(ToR:CR)_ ^¶^	5/40 (12.5)		
	ToR-A ^¶^	3/40 (7.5)		
	ToS-B + ToR-B ^&^	9/40 (22.5)		
	ToS-B ^¶^	0/40 (0)		
	ToR-B ^¶^	14/40 (35)		
ToSRV-A_(ToR:IRD)_ + ToRMV	ToS-A_(ToR:IRD)_ + ToR-A ^&^	1/40 (2.5)	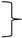	**
	ToS-A_(ToR:IRD)_ ^¶^	6/40 (15)		
	ToR-A ^¶^	10/40 (25)		
	ToS-B + ToR-B ^&^	1/40 (2.5)		
	ToS-B ^¶^	1/40 (2.5)		
	ToR-B ^¶^	12/40 (30)		
ToSRV-A_(ToR:CR+IRD)_ + ToRMV	ToS-A_(ToR:CR+IRD)_ + ToR-A ^&^	6/38 (15.8)	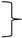	**
	ToS-A_(ToR:CR+IRD)_ ^¶^	4/38 (10.5)		
	ToR-A ^¶^	5/38 (13.1)		
	ToS-B + ToR-B ^&^	3/38 (7.9)		
	ToS-B ^¶^	0/38 (0)		
	ToR-B ^¶^	15/38 (39.5)		
ToSRV_(wt)_ + ToRMV	ToS-A + ToR-A ^&^	4/40 (10)	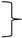	**
	ToS-A ^¶^	7/40 (17.5)		
	ToR-A ^¶^	11/40 (27.5)		
	ToS-B + ToR-B ^&^	4/40 (10)		
	ToS-B ^¶^	1/40 (2.5)		
	ToR-B ^¶^	12/40 (30)		

* For simplicity, treatments are named based on DNA-A; wild-type DNA-B components were always inoculated together with their cognate DNA-A components. ^$^ Number of infected plants/number of inoculated plants (percentage of infected plants), confirmed by PCR with virus-specific primers (single infection) or by RCA-RFLP (mixed infection) at 28 days after inoculation. Results correspond to the sum of four independent experiments. See Supplementary Table S1 for results of each one of the four experiments. Statistical analysis (Wilcoxon rank sum test) refers to the comparison between ToSRV_(wt)_ (first line of the table) and the treatment indicated (ns, non-significant; **, significant). See Supplementary Table S2 for details. ^#^ Plants inoculated with each virus in single infection. ^&^ Plants inoculated with both viruses in which the DNA-A or DNA-B of both viruses were detected. ^¶^ Plants inoculated with both viruses in which the DNA-A or DNA-B of only one of the two viruses was detected.

## Data Availability

All data generated or analyzed during this study are included in this published article (and its Supplementary Materials Files).

## Supplementary Materials

The following supporting information can be downloaded at: https://www.mdpi.com/article/10.3390/v15102074/s1, Figure S1: Symptoms in tomato plants biolistically inoculated with tomato rugose mosaic virus (ToRMV) and tomato severe rugose virus (ToSRV_(wt)_, ToSRV-A_(ToR:CR)_, ToSRV-A_(ToR:IRD)_ and ToSRV-A_(ToV:CR+IRD)_) infectious clones in single and mixed infections; Figure S2: Specific detection of DNA-A and DNA-B components of tomato rugose mosaic virus (ToRMV) and tomato severe rugose virus (ToSRV) in mixed infection by RCA-RFLP.; Figure S3: Weblogo describing the multiple alignment of the common region (CR) and Rep partial nucleotide sequences of tomato severe rugose virus (ToSRV) isolates; Table S1: Infectivity of tomato severe rugose virus (ToSRV) clones in single infection and in mixed infection with tomato rugose mosaic virus (ToRMV); Table S2: Infectivity statistics of clones of tomato severe rugose virus (ToSRV) in single infection and mixed infection with tomato rugose mosaic virus (ToRMV).
