# Supplementary material for: Specific Nucleotides in the Common Region of the Begomovirus Tomato Rugose Mosaic Virus (ToRMV) Are Responsible for the Negative Interference over Tomato Severe Rugose Virus (ToSRV) in Mixed Infection
Source: Viruses. 2023 Oct 11;15(10):2074. doi: 10.3390/v15102074 (PMC10611410; doi:10.3390/v15102074)
Supplement: Supplementary file 1 [file viruses-15-02074-s001.zip › viruses-2619457-supplementary.pdf]

Supplementary Table S1. Infectivity of tomato severe rugose virus (ToSRV) clones in single infection and in mixed infection with tomato rugose mosaic virus (ToRMV).

Treatment[*]	Genomic component	Infected plants[$]			
		Exp. I	Exp. II	Exp. III	Exp. IV
ToSRV-A$_{(wt)}$	ToS-A$_{(wt)}$[#]	3/10 (30)	4/10 (40)	4/10 (40)	5/10 (50)
ToRMV-A	ToR-A[#]	4/10 (40)	3/10 (30)	3/10 (30)	4/10 (40)
ToSRV-A$_{(ToR:CR)}$	ToS-A$_{(ToR:CR)}$[#]	6/10 (60)	1/10 (10)	7/9 (78)	5/10 (50)
ToSRV-A$_{(ToR:IRD)}$	ToS-A$_{(ToR:IRD)}$[#]	6/10 (60)	2/10 (20)	10/10 (100)	5/10 (50)
ToSRV-A$_{(ToR:CR+IRD)}$	ToS-A$_{(ToR:CR+IRD)}$[#]	7/10 (70)	3/10 (30)	7/10 (70)	3/10 (30)
ToSRV-A$_{(ToR:CR)}$/ToRMV-A	ToS-A$_{(ToR:CR)}$+ToR-A[&]	2/10 (20)	5/10 (50)	7/10 (70)	1/10 (10)
	ToS-A$_{(ToR:CR)}$[¶]	0/10 (0)	1/10 (10)	2/10 (10)	2/10 (20)
	ToR-A[¶]	0/10 (0)	1/10 (10)	1/10 (10)	1/10 (10)
	ToS-B+ToR-B[&]	0/10 (0)	0/10 (0)	3/10 (30)	2/10 (20)
	ToS-B[¶]	0/10 (0)	0/10 (0)	0/10 (0)	0/10 (0)
	ToR-B[¶]	2/10 (20)	7/10 (70)	3/10 (30)	2/10 (20)
ToSRV-A$_{(ToR:IRD)}$/ToRMV-A	ToS-A$_{(ToR:IRD)}$+ToR-A[&]	0/10 (0)	0/10 (0)	1/10 (10)	0/10 (0)
	ToS-A$_{(ToR:IRD)}$[¶]	2/10 (20)	1/10 (10)	1/10 (10)	2/10 (20)
	ToR-A[¶]	3/10 (30)	3/10 (30)	1/10 (10)	3/10 (30)
	ToS-B+ToR-B[&]	1/10 (10)	0/10 (0)	0/10 (0)	0/10 (0)
	ToS-B[¶]	0/10 (0)	1/10 (10)	0/10 (0)	0/10 (0)
	ToR-B[¶]	4/10 (40)	3/10 (30)	1/10 (10)	4/10 (40)
ToSRV-A$_{(ToR:CR+IRD)}$/ToRMV-A	ToS-A$_{(ToR:CR+IRD)}$+ToR-A[&]	2/10 (20)	1/10 (10)	3/9 (33)	0/9 (0)
	ToS-A$_{(ToR:CR+IRD)}$[¶]	1/10 (10)	2/10 (20)	0/9 (0)	1/9 (11)
	ToR-A[¶]	1/10 (10)	2/10 (20)	1/9 (11)	1/9 (11)
	ToS-B+ToR-B[&]	1/10 (10)	0/10 (0)	2/9 (22)	0/9 (0)
	ToS-B[¶]	0/10 (0)	0/10 (0)	0/9 (0)	0/9 (0)
	ToR-B[¶]	3/10 (30)	5/10 (50)	3/9 (33)	4/9 (44)
ToSRV-A$_{(wt)}$/ToRMV-A	ToS-A$_{(wt)}$+ToR-A[&]	2/10 (20)	0/10 (0)	2/10 (20)	0/10 (0)
	ToS-A$_{(wt)}$[¶]	2/10 (20)	2/10 (20)	1/10 (10)	2/10 (20)
	ToR-A[¶]	1/10 (10)	3/10 (30)	4/10 (40)	3/10 (30)
	ToS-B+ToR-B[&]	1/10 (10)	2/10 (20)	1/10 (10)	0/10 (0)
	ToS-B[¶]	0/10 (0)	1/10 (10)	0/10 (0)	0/10 (0)
	ToR-B[¶]	4/10 (40)	2/10 (20)	4/10 (40)	2/10 (20)

[*] For simplicity, treatments are named based on the DNA-A; wild-type DNA-B components were always inoculated together with their cognate DNA-A components.

[$] Number of infected plants/number of inoculated plants (percentage of infected plants), confirmed by PCR with virus-specific primers (single infection) or RCA-RFLP (mixed infection) at 28 days after inoculation. Four independent treatments were carried out.

[#] Plants inoculated with each virus in single infection.

[&] Plants inoculated with both viruses in which DNA-A or DNA-B of both viruses were detected.

[¶] Plants inoculated with both viruses in which DNA-A or DNA-B of only one of the two viruses was detected.

Supplementary Table S2. Infectivity statistics of clones of tomato severe rugose virus (ToSRV) in single infection and mixed infection with tomato rugose mosaic virus (ToRMV).

**Comparison**	**Wilcoxon rank sum test**	**Student's *t*-test**
Single infection		
$ToSRV_{(wt)}$ *vs* ToRMV	p = 0.4292 ns	p = 0.36 ns
$ToSRV_{(wt)}$ *vs* $ToSRV_{(ToR:CR)}$	p = 0.3807 ns	p = 0.62 ns
$ToSRV_{(wt)}$ *vs* $ToSRV_{(ToR:IRD)}$	p = 0.3572 ns	p = 0.37 ns
$ToSRV_{(wt)}$ *vs* $ToSRV_{(ToR:CR+IRD)}$	p = 0.8809 ns	p = 0.46 ns
Mixed infection		
$ToSRV_{(wt)}$ *vs* $ToSRV_{(wt)}$ + ToRMV	p = 0.0275**	p = 0.007**
$ToSRV_{(wt)}$ *vs* ToSRV-$A_{(ToR:CR)}$ + ToRMV	p = 1 ns	p = 0.87 ns
$ToSRV_{(wt)}$ *vs* $ToSRV_{(ToR:IRD)}$ + ToRMV	p = 0.0256**	p = 0.006**
$ToSRV_{(wt)}$ *vs* $ToSRV_{(ToR:CR+IRD)}$ + ToRMV	p = 0.0408**	p = 0.02**

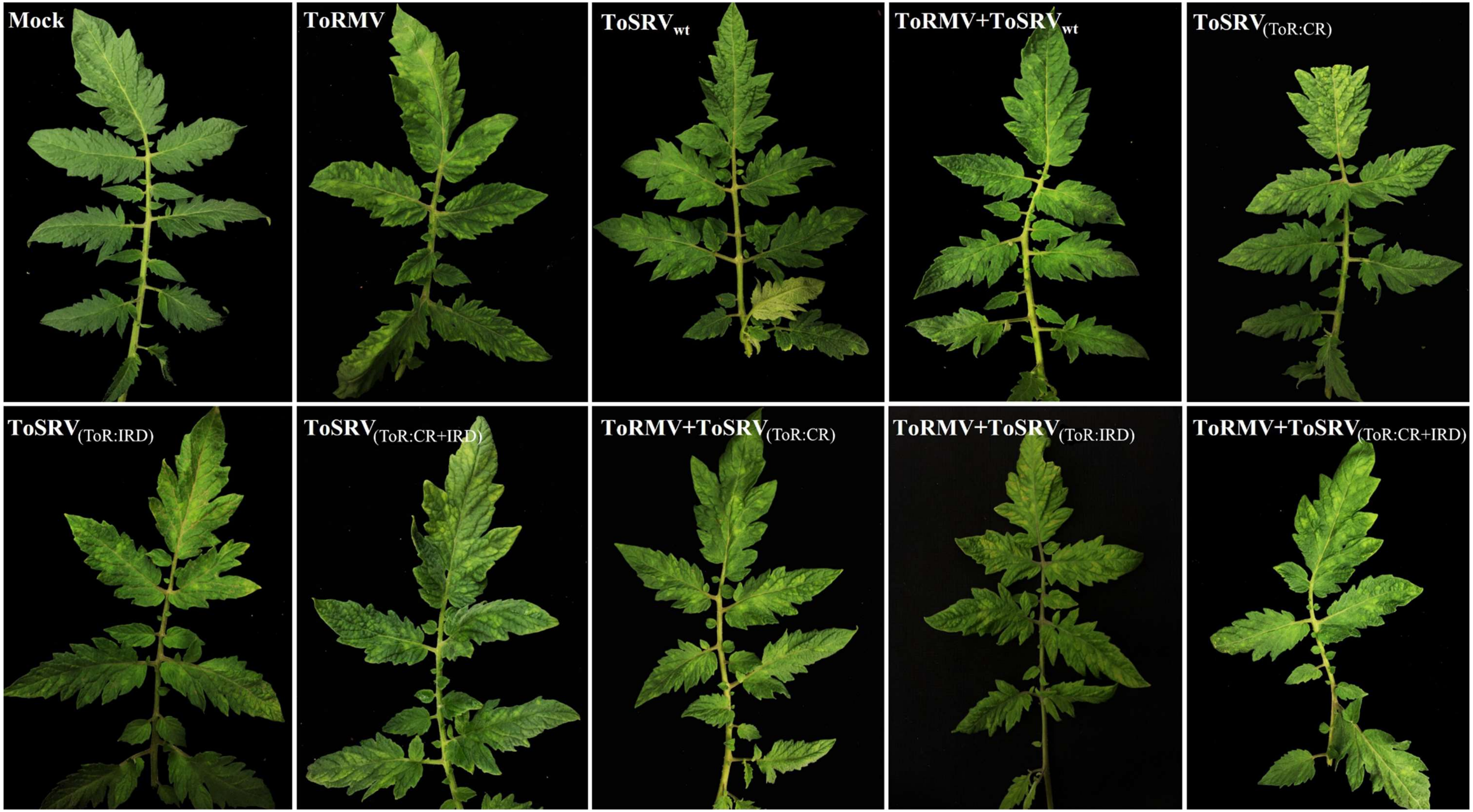

Supplementary Figure S1. Symptoms in tomato plants biolistically inoculated with tomato rugose mosaic virus (ToRMV) and tomato severe rugose virus (ToSRV$_{(wt)}$, ToSRV-A$_{(ToR:CR)}$, ToSRV-A$_{(ToR:IRD)}$ and ToSRV-A$_{(ToV:CR+IRD)}$) infectious clones in single and mixed infection, as indicated. All images obtained at 28 days after inoculation.

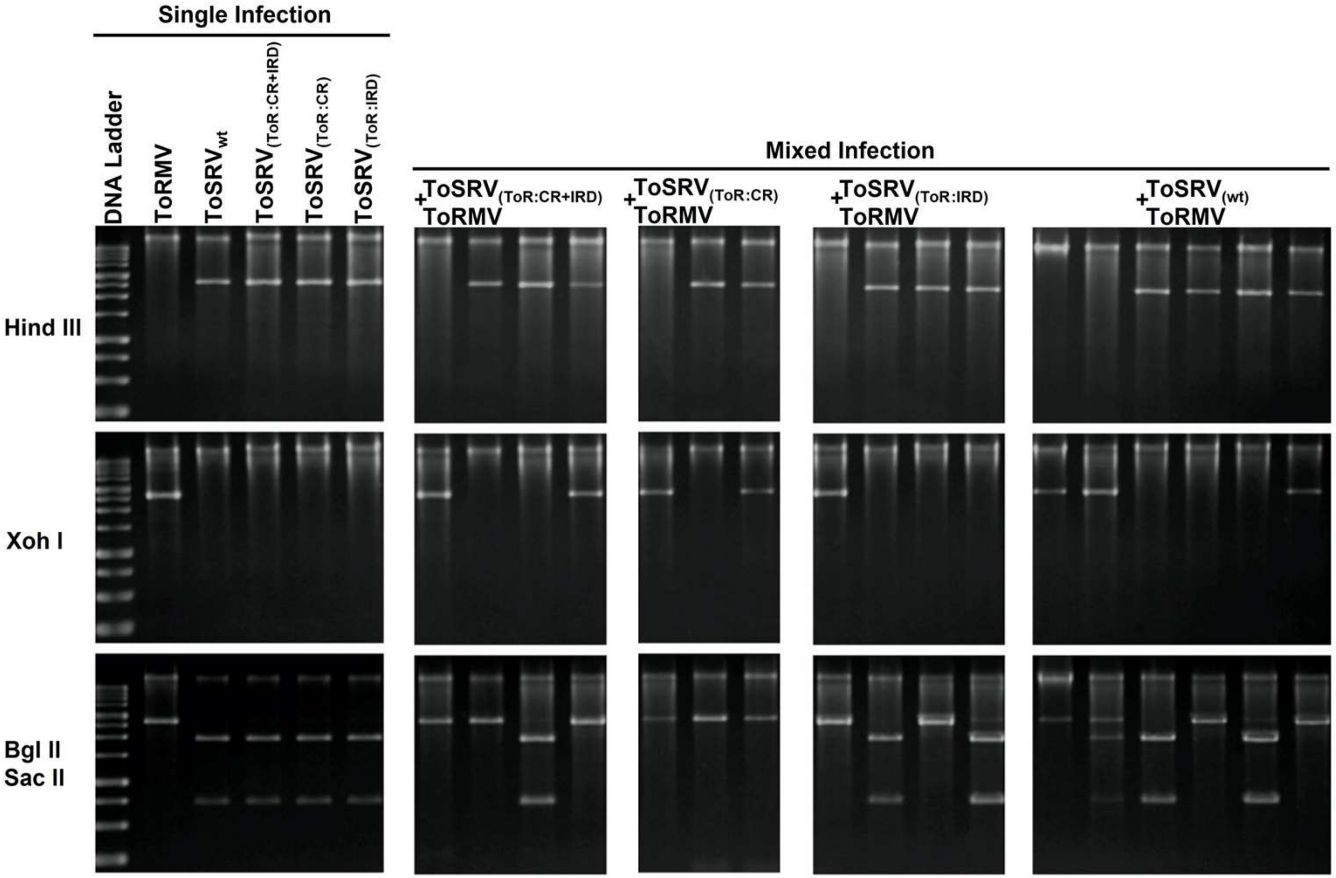

Supplementary Figure S2. Specific detection of DNA-A and DNA-B components of tomato rugose mosaic virus (ToRMV) and tomato severe rugose virus (ToSRV) in mixed infection by RCA-RFLP. Digestion with HindIII enables the detection of ToSRV DNA-A, generating a fragment of approximately 2,600 nucleotides (nt). Digestion with XhoI enables the detection of ToRMV DNA-A, generating a fragment of approximately 2,600 nt. Digestion with BglII+SacII enables the detection and differentiation of DNA-B components, generating fragments of 1,800 and 800 nt for ToSRV DNA-B and approximately 2,600 nt for ToRMV DNA-B. Detection of ToRMV and ToSRV in single infection shown as controls. M, size marker (1 kb plus DNA ladder, Kasvi).

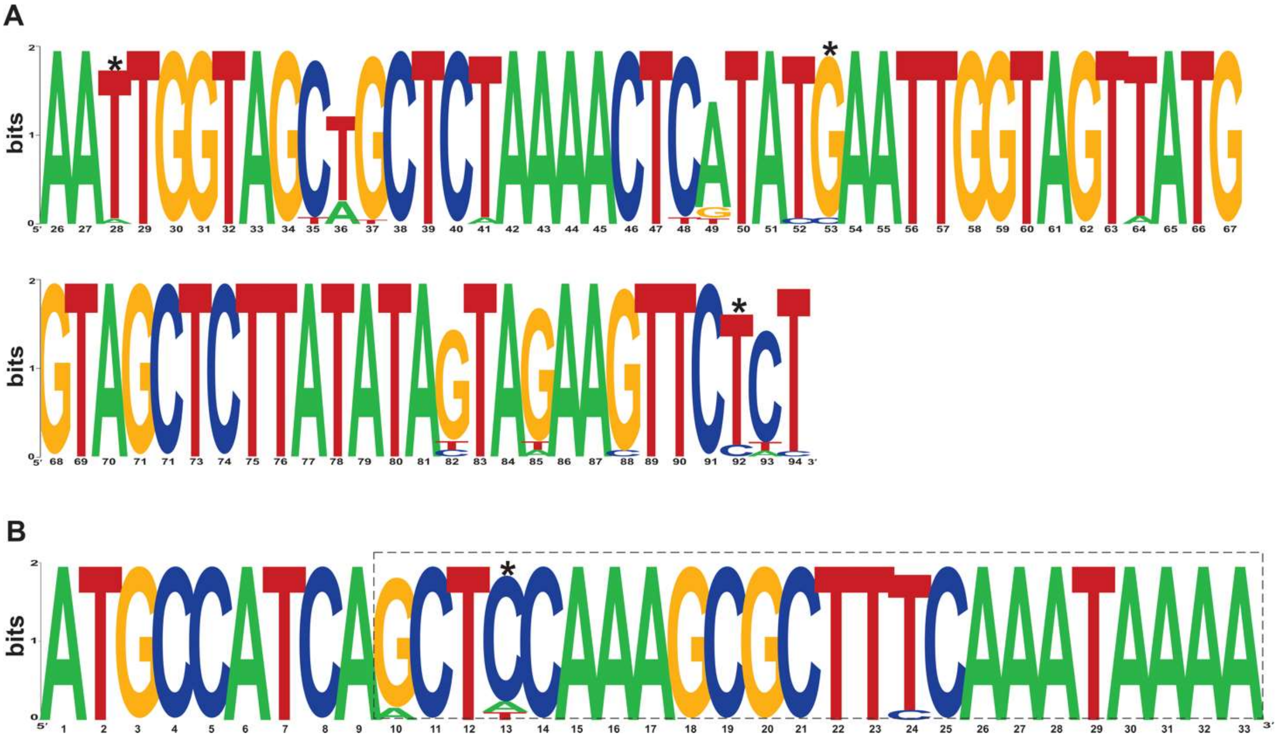

Supplementary Figure S3. Weblogo (created using the WebLogo web server at weblogo.threeplusone.com) describing the multiple alignment of the common region (CR) and Rep partial nucleotide sequences of tomato severe rugose virus (ToSRV) isolates. **A.** Partial alignment of CR nucleotide sequences including the divergent positions 1-5. **B.** Partial alignment of Rep nucleotide sequences including the IRD domain delimited by dashed lines. The asterisks correspond to the altered nucleotides for the construction of the ToSRV-A$_{(ToR:IRD)}$ and ToSRV-A$_{(ToR:CR+IRD)}$ clones. The y-axis represents the bit score, where 2 means 100% conservation. The x-axis displays the nucleotide position in the multiple sequence alignment. The height of the letters indicates the level of conservation at that specific position.
